# Area-specific autoencoder spatiotemporal graph neural networks for opioid overdose death prediction

**DOI:** 10.1093/jamiaopen/ooag063

**Published:** 2026-04-29

**Authors:** Xianhui Chen, Changchang Yin, John V Myers, Brandon Slover, Neena Thomas, Charles Marks, Joanne Kim, Soledad Fernández, Penn Whitley, Naleef Fareed, Ping Zhang

**Affiliations:** Computer Science and Engineering, The Ohio State University, Columbus, OH, United States; Department of Biomedical Informatics, The Ohio State University, Columbus, OH, United States; Department of Biomedical Informatics, The Ohio State University, Columbus, OH, United States; Center for Biostatistics, The Ohio State University, Columbus, OH, United States; Center for Biostatistics, The Ohio State University, Columbus, OH, United States; Center for Biostatistics, The Ohio State University, Columbus, OH, United States; Millennium Health, San Diego, CA, United States; Department of Biomedical Informatics, The Ohio State University, Columbus, OH, United States; Center for Biostatistics, The Ohio State University, Columbus, OH, United States; Department of Biomedical Informatics, The Ohio State University, Columbus, OH, United States; Center for Biostatistics, The Ohio State University, Columbus, OH, United States; Millennium Health, San Diego, CA, United States; Department of Biomedical Informatics, The Ohio State University, Columbus, OH, United States; Computer Science and Engineering, The Ohio State University, Columbus, OH, United States; Department of Biomedical Informatics, The Ohio State University, Columbus, OH, United States

**Keywords:** opioid overdose death prediction, spatiotemporal graph neural network, public health, deep learning

## Abstract

**Background:**

Ohio has been severely impacted by the opioid crisis, with opioid overdose (OD) death rates exceeding national averages. Accurate OD death prediction supports proactive prevention and treatment allocation. Existing methods often focus on ZIP Code Tabulation Area (ZCTA)–level prediction for small-area resource allocation; however, performance at this resolution is poor due to substantial fluctuations in OD death counts, which introduce noise. This raises a critical methodological question: what is the optimal population threshold for OD death prediction that balances predictive accuracy with geographic resolution?

**Methods:**

We perform a theoretical analysis of variance and error bounds to establish the minimal population required for robust prediction. Building on this analysis, we propose an Area-specific Autoencoder Spatiotemporal Graph Neural Network (AAE-STGNN) framework for OD death count prediction using urine drug test (UDT) data as dynamic features and Social Determinants of Health (SDoH) as static features. The framework consists of two key components: (1) an *Area-specific Autoencoder (AAE)*, which learns latent spatial representations while incorporating the minimal population threshold, and (2) a *Spatiotemporal Graph Neural Network (STGNN)*, which models geographic adjacency between areas and dynamic features across time.

**Results:**

Empirical evaluations demonstrate that AAE-STGNN outperforms state-of-the-art (SOTA) approaches, achieving improved accuracy and robustness. We also provide the OD death count trend estimation to support public health decision-making.

**Conclusions:**

These findings underscore the importance of selecting an optimal spatial granularity and leveraging spatiotemporal modeling techniques for data-driven public health surveillance and targeted intervention in the opioid crisis.

## 1 Introduction

Ohio remains one of the states most severely affected by the opioid epidemic.^1^^,^^2^ In 2024, Ohio’s OD death rate was 1.18 times the national average (19.29 versus 16.30 per 100,000).^3^ According to the 2023 Ohio Unintentional Drug Overdose Report,^4–6^ the crisis has been exacerbated by the widespread availability of synthetic opioids such as fentanyl, which accounted for 81% of OD deaths that year. Predictive algorithms have emerged as promising tools for forecasting opioid-related mortality, enabling health agencies to proactively allocate resources, guide targeted interventions, and rapidly respond to emerging overdose hotspots before severe surges occur, and have been noted as cost-effective and accessible complements to traditional surveillance approaches.^7^^,^^8^

However, existing approaches face three major challenges. (1) Previous predictive efforts highlight the importance of forecasting outcomes at the ZCTA-level^9–11^ to support precise policy decisions and resource allocation. Our preliminary analysis, however, indicates that ZCTA-level prediction of OD deaths in Ohio performs poorly, yielding a 162.55% relative difference between predicted and observed values, as measured by the Symmetric Mean Absolute Percentage Error (SMAPE). At this spatial resolution, even small absolute errors translate into disproportionately large percentage deviations. For example, mispredicting by just one death in a community of four individuals results in a 25% deviation, whereas the same absolute error in a population of 10,000 corresponds to only 0.01%. (2) Conventional models that assume independence across regions or time fail to capture strong spatiotemporal coherence, resulting in biased or unstable predictions. (3) Existing approaches often fail to incorporate important indicators of social environments, hindering model generalization across communities and ultimately constraining predictive accuracy and real-world applicability.^12^

In this study, we first provide a theoretical analysis of the minimal population required under various confidence levels (i.e., variance) and, based on this analysis, reformulate the OD prediction problem at the county level. We further identify minimal population thresholds that balance predictive performance and geospatial resolution. Building on this reformulation, we propose a novel framework, AAE-STGNN, for geospatial representation and OD prediction. Specifically, to mitigate the substantial volatility, we perform augmented feature representation learning using an AAE, in which neighboring ZCTAs are randomly aggregated into synthetic regions that exceed a population threshold. For each augmented region, we recompute comprehensive features, including UDT indicators, SDoH, and geospatial lag variables, and generate latent representations for prediction. In addition, we capture spatial and temporal dependencies using a STGNN, which leverages geographic adjacency and historical trends to enhance predictive accuracy.

To demonstrate the effectiveness of the proposed AAE-STGNN, we conducted experiments on OD death counts in Ohio using UDT and SDoH data. The results show that our model outperforms SOTA approaches, achieving a 15.70% improvement in SMAPE. In addition, we provide trend estimation based on the predicted OD death counts relative to the previous prediction window, achieving 1.03% higher accuracy than SOTA. Our approach enables more precise and proactive identification of high-risk communities, supporting targeted interventions for OD death prevention.

Our contributions can be summarized as follows:

- We propose a novel AAE-STGNN framework for county-level OD death prediction using UDT and SDoH data, jointly modeling spatial dependencies among neighboring counties and temporal dynamics in historical observations.
- We present a theoretical analysis of OD death fluctuations to derive a minimal population threshold, which guides the design of an AAE for learning latent, area-specific representations and motivates aggregating neighboring ZCTAs into synthetic regions for model training.
- Experimental results on Ohio OD death counts demonstrate that the model outperforms SOTA approaches by over 15.70% in SMAPE and 2.23% in accuracy for trend estimation, highlighting the effectiveness of the proposed framework.

## 2 Related work

### 2.1 Small-area opioid-related analysis

Some existing studies focus on ZCTA-level or even finer spatial units for OD death prediction to support policy decisions and resource allocation. For example, Schell et al.^13^ applied LASSO, followed by variable-importance rankings from a random forest model, to perform census block level prediction in Rhode Island, where each block typically contains 600–3,000 residents. Bauer et al.^9^ developed Bayesian spatiotemporal dynamic models to predict OD death across Massachusetts’ 537 ZCTAs, evaluating predictive performance using a one-year–ahead forecasting approach. Srinivasan et al.^14^ employed Local Indicators of Spatial Association (LISA) cluster analyses using raw incidence rates and Empirical Bayes–smoothed rates to identify high-risk ZCTAs in Massachusetts.

However, these methods often suffer from poor predictive performance due to the high variance associated with small population sizes.^15^ Motivated by this challenge, our study investigates the minimal population size necessary to ensure reliable OD death prediction.

### 2.2 OD death prediction

A growing body of work has explored statistical and machine learning approaches for predicting OD deaths. In response to the national rise in heroin- and fentanyl-related fatalities, Ferris et al.^16^ used multivariate logistic regression with a 6-month lookback window to predict individual-level OD death among adults in Maryland. Sumner et al.^8^ employed a LASSO regression model to generate near real-time weekly national-level predictions of OD deaths for 2018–2019 using multiple proxy data sources. Matero et al.^17^ introduced TROP (Transformer for Opioid Prediction), a community-level forecasting model that incorporates community-specific social media language features along with historical OD death data to predict future changes in opioid-related deaths by county.

Despite their contributions, these methods typically overlook the spatial correlations across neighboring communities as well as the temporal dependencies embedded in historical OD death count trends. Both factors are fundamental to overdose dynamics: spatial spillover arises from shared drug supply networks and socioeconomic conditions, while temporal continuity reflects the persistence of local risk environments and reporting patterns. Ignoring these dependencies often results in unstable or biased estimates. Accordingly, we explicitly incorporate both spatial and temporal structures to capture these underlying processes and improve prediction performance.

## 3 Theoretical analysis and problem formulation

Our preliminary experiments show that ZCTA-level prediction of OD death counts in Ohio performs poorly, yielding a 162.55% SMAPE. Accurate OD death count prediction at fine geographic resolutions is fundamentally constrained by data sparsity. To address this limitation, we analyze the impact of limited population size on prediction reliability and develop a theoretical analysis that quantifies how population size affects achievable prediction performance. This analysis is critical for identifying the minimal population thresholds needed to produce stable and actionable predictions at the ZCTA-level.

To characterize the statistical uncertainty in OD death count prediction at the ZCTA-level, we examine the signal-to-noise ratio (SNR) and SMAPE.

### 3.1 Assumptions

Assumption 3.1(Randomness of OD Counts). *For a given area, OD death counts Y are considered random variables.*

Assumption 3.2(Large Population Approximation). *Let n denote the population size and p denote the underlying OD death risk for an individual in that unit. Then when np is large, Y can be approximated by a normal distribution:*
 

Y∼N(np,np).

Assumption 3.3(Small Population Approximation). *When*  np≪1*, Y can be approximated by a Bernoulli distribution:*
 

Y∼Bernoulli(np),

*With predicted value* ${\hat{y}}_{i}=np$.

### 3.2 SNR Analysis for Large Area

For large area, we assume the OD death follows the normal approximation Y∼N(np,np).

**SNR Analysis:** The expected overdose death count and variance are


(1) E[Y]=np,

(2) Var(Y)=np,

Which leads to a standard deviation of


(3) $$\text{Std}(Y)=\sqrt{np}.$$

The SNR is therefore


(4) $$\text{SNR}=\frac{\text{Signal}}{\text{Noise}}=\frac{E[Y]}{\text{Std}(Y)}=\frac{np}{\sqrt{np}}=\sqrt{np}.$$

**SMAPE Analysis:** For area *i*, SMAPE is defined as


(5) $${\text{SMAPE}}_{i}=\frac{2|y_{i}-{\hat{y}}_{i}|}{\left|y_{i}|+|{\hat{y}}_{i}\right|},$$

Where $y_{i}$ is the observed OD count and ${\hat{y}}_{i}$ is the predicted count. Even if the model is perfectly calibrated, i.e., ${\hat{y}}_{i}=E[Y]=np$, stochastic fluctuations in $y_{i}$ will produce non-zero error. The expected absolute deviation is


(6) $$E[|Y-np|]=\sqrt{\frac{2np}{\pi }},$$

So that the expected SMAPE becomes


(7) $$E[{\text{SMAPE}}_{i}]\approx \frac{2\cdot \sqrt{\frac{2np}{\pi }}}{2np}=\sqrt{\frac{2}{\pi np}}.$$

### 3.3 SNR analysis for small area

For small populations or rare events, the OD death is usually 0 or 1. When np≪1, we can approximate Y∼Bernoulli(np) with ${\hat{y}}_{i}=np$.

**SNR Analysis:** The expected overdose death count and variance are


(8) E[Y]=np,

(9) Var(Y)=np(1−p)≈np(1−np),

Leading to a standard deviation of


(10) $$\text{Std}(Y)=\sqrt{np(1-np)}.$$

The SNR is therefore


(11) $$\text{SNR}=\frac{\text{Signal}}{\text{Noise}}=\frac{E[Y]}{\text{Std}(Y)}=\frac{np}{\sqrt{np(1-np)}}.$$

**SMAPE Analysis:** The expected SMAPE is


(12) $$E[{\text{SMAPE}}_{i}]=(np)\cdot \frac{2(1-np)}{1+np}+(1-np)\cdot 2$$

(13) $$=2\cdot \frac{1+np-{\left(np\right)}^{2}}{1+np}.$$

### 3.4 Relationship between confidence level over population size

**Figure 1.**
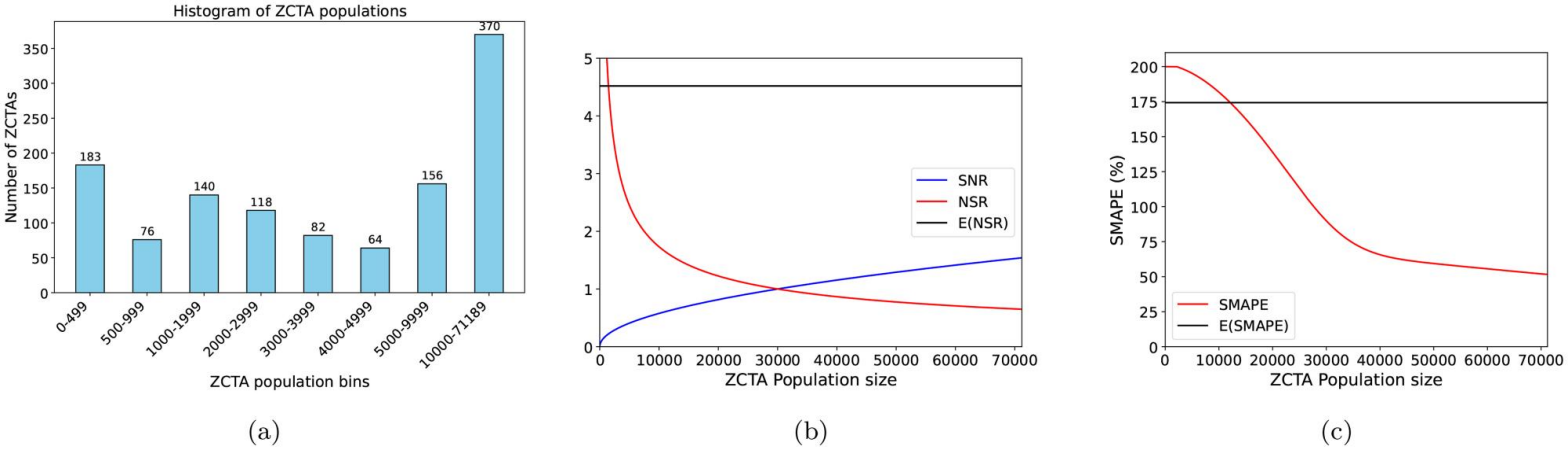
Theoretical analysis for population threshold derivation. (a) Population size distribution across Ohio ZCTAs. (b) Population-dependent SNR and noise-to-signal ratios (NSR), with E(NSR) indicating the expected NSR at the average ZCTA population. (c) SMAPE across population groups, where E(SMAPE) denotes the expected SMAPE at the average ZCTA population.

Across Ohio, ZCTA populations show substantial variability, ranging from a minimum of 12 to a maximum of 71,189, with a mean of 9,802 and a median of 3,899, as shown in Figure 1(a). Assuming an annual overdose death rate of 4 per 10,000 individuals, the corresponding probability is


(14) $$p=\frac{\frac{4}{10,000}}{12}=\frac{1}{30,000}.$$

For typical Ohio ZCTAs, the average SNR is about 4.5, indicating that more than 80% of the observed OD counts are attributable to statistical noise rather than true signal (Figure 1(b)). This high noise level poses a significant challenge for predictive modeling, as it obscures underlying patterns and limits achievable accuracy at this fine spatial and temporal resolution.

Figure 1(c) shows the SMAPE change over the population, demonstrating that prediction at very small spatial scales is dominated by noise, reinforcing the need to select an aggregation level that balances predictive performance with actionable geographic resolution.

**Conclusion:** Based on the SNR and SMAPE analysis, a minimal population threshold of 30,000 emerges as the optimal geospatial resolution, effectively mitigating noise while preserving geographic granularity that is actionable.

### 3.5 Problem formulation

**Figure 2.**
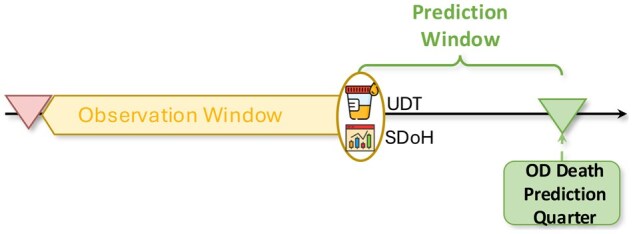
Study design. The observation window contains historical UDT and SDoH data. The prediction window accounts for the time required for data collection by public health agencies. For example, we use UDT data from Q1–Q3 2019 together with SDoH scores to predict OD death counts in the target quarter, Q1 2020.

Based on the minimal population threshold analysis described above, we perform quarterly county-level OD death prediction for Ohio. Among the state’s 88 counties, 72 (≈ 82%) meet or exceed the 30,000-person threshold. Although not all counties satisfy the requirement, the county level provides the smallest geographic unit for which the vast majority of regions have adequate population size, offering a resolution that may support policy planning at the regional level. Considering the time lag in data collection by public health agencies, we adopt a prediction window of one quarter (three months), meaning the model forecasts OD deaths for the quarter following the prediction window. The observation window consists of historical data from prior quarters used as input. Figure 2 illustrates the study design for OD death prediction. The model uses data from the observation window to predict OD deaths in the target quarter.

Predictions for counties below the 30,000 population threshold should be interpreted as lower-confidence estimates. While absolute count predictions in these areas could be less accurate, the outputs can still be useful for qualitative trend monitoring, regional situational awareness, and early warning when interpreted alongside neighboring counties or regional aggregates.

## 4 Methodology

**Figure 3.**
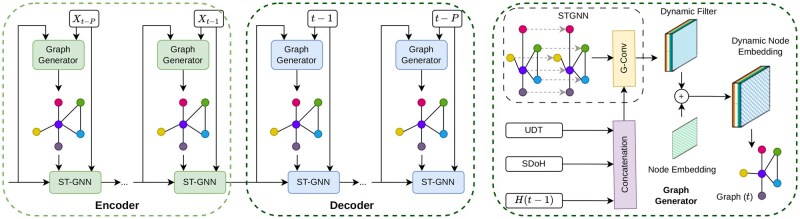
Overview of the proposed model framework. The AAE comprises an encoder–decoder architecture for learning informative latent representations. The STGNN is integrated into the AAE, where historical UDT, SDoH, and spatial-lag features are provided to the STGNN to extract both temporal and spatial patterns.

Figure 3 shows the architecture of the proposed AAE-STGNN.

### 4.1 Data preprocessing

The construction of the analytic dataset integrates information on OD death, drug-use patterns, and SDoH. All data sources were harmonized at a quarterly resolution and aligned over a shared study period. A summary of included variables is provided in Table 5 and detailed descriptions of the outcome and exposure data components are listed in Appendix D, available as supplementary data at [*JAMIA Open*] online.

### 4.2 Feature representation learning with an area-specific autoencoder

This component focuses on feature representation learning to capture the underlying spatial and contextual patterns associated with OD deaths. To address the sparsity of UDT data and improve the model’s ability to learn stable associations between UDT patterns and OD deaths while reducing overfitting, we introduce a data augmentation strategy that randomly aggregates neighboring ZCTAs into synthetic areas with sufficiently dense UDT signals. This ensures that each generated area contains a sufficient population size to produce stable and meaningful patterns.

For each synthetic area, we recompute all feature variables to reflect its aggregated characteristics and sum the corresponding overdose deaths as the outcome label. By varying the population threshold *n*, we generate multiple augmented datasets to examine the trade-offs across different spatial scales. We then train an area-specific autoencoder on these datasets to encode each area’s characteristics into a fixed-length latent vector z.

**AAE:** Let $x_{i}$ denote the input features for area *i*, and $z_{i}$ its latent embedding. The encoder maps $x_{i}$ to a latent distribution:


(15) $$\begin{array}{cc} \mu_{i},\sigma_{i}^{2}=\text{Encoder}(x_{i}), & \\ z_{i}∼N(\mu_{i},\sigma_{i}^{2}), & \end{array}$$

Where $\mu_{i}$ and $\sigma_{i}^{2}$ represent the mean and variance of the latent distribution for area *i*, respectively.

The decoder reconstructs the input:


(16) $${\hat{x}}_{i}=\text{Decoder}(z_{i})$$

The reconstruction loss is the mean squared error (MSE):


(17) $$L_{\text{recon}}=\frac{1}{N}\sum_{i}\|x_{i}-{\hat{x}}_{i}{\|}^{2}$$

Where *N* is the number of areas.

For areas with labels $y_{i}$, the supervised prediction loss is:


(18) $${\hat{y}}_{i}=f_{\text{pred}}(z_{i}),$$

(19) $$L_{\text{sup}}=\frac{1}{N_{\text{labeled}}}\sum_{i\in \text{labeled}}\|y_{i}-{\hat{y}}_{i}{\|}^{2}$$

The total loss for AAE is:


(20) $$L_{\text{AAE}}=L_{\text{recon}}+\gamma L_{\text{sup}},$$

Where γ controls the trade-off between reconstruction and supervised prediction.

### 4.3 STGNN for multi-modal spatiotemporal modeling

We also incorporate spatial and temporal dependencies into the prediction framework using a STGNN. The STGNN is designed to leverage both spatial adjacency and temporal dynamics in OD death data. By explicitly modeling geographic connectivity and temporal dynamics, the proposed network effectively captures spillover effects and shared progression patterns across counties. The Long Short-Term Memory (LSTM) component enhances robustness by learning temporal trends even in regions with sparse observations. In addition, the graph convolution module adaptively learns importance weights for both neighboring regions and key input features, enabling the model to capture spatial spillover effects. By aggregating information from adjacent areas—including residents’ substance use pattern (UDT) and SDoH—the model accounts for how these factors in neighboring regions influence OD death count, while simultaneously identifying the most influential features driving OD deaths. Through this framework, we integrate rich feature representations with spatiotemporal modeling, enabling more accurate overdose death prediction and a principled evaluation of predictive performance across different geographic resolutions.

Let $G_{t}=(V,E)$ denote the graph of regions at time *t*, where *V* is the set of regions (nodes) and *E* encodes adjacency. Each node $v_{i}$ has features $x_{i}^{t}$ obtained from the AAE latent vector.

**Graph Convolution:** For each node, two Graph Convolution (GCN) layers update node embeddings sequentially:


(21) $$h_{i}^{\left(1\right)}=\sigma (\sum_{j\in N(i)\cup \{i\}}\frac{1}{\sqrt{d_{i}d_{j}}}W^{\left(0\right)}x_{j}),$$

(22) $$h_{i}^{\left(2\right)}=\sigma (\sum_{j\in N(i)\cup \{i\}}\frac{1}{\sqrt{d_{i}d_{j}}}W^{\left(1\right)}h_{j}^{\left(1\right)}),$$

Where $x_{j}$ is AAE latent vector, N(i) denotes neighbors of node *i*, $d_{i}$ is node degree, $W^{\left(0\right)},W^{\left(1\right)}$ are learnable weight matrices for the first and second layers, and σ is an activation function (ReLU).

**Temporal Modeling (LSTM):** The GCN outputs $h_{i}^{\left(2\right)}$ for each area across time are fed into an LSTM to capture temporal dynamics:


(23) $$s_{i}^{\left(t\right)}=\text{LSTM}(h_{i}^{\left(2,t\right)},s_{i}^{\left(t-1\right)})$$

**Prediction and Loss:** The final prediction of OD death counts for area *i* at time *t* and the training MSE loss are defined as:


(24) $${\hat{y}}_{i}^{\left(t\right)}=W_{\text{out}}s_{i}^{\left(t\right)}+b,$$

(25) $$L_{\text{STGNN}}=\frac{1}{N}\sum_{i=1}^{N}{\left(y_{i}^{\left(t\right)}-{\hat{y}}_{i}^{\left(t\right)}\right)}^{2},$$

Where $W_{\text{out}}$ and *b* are learnable parameters.

## 5 Experiment setup

### 5.1 Evaluation metrics

We use multiple evaluation metrics—SMAPE, Mean Absolute Error (MAE), and Root Mean Squared Error (RMSE)—to assess both absolute and relative prediction errors, capturing different aspects of predictive performance. Lower values for all metrics indicate better predictions. The detailed description is provided in Appendix A, available as supplementary data at [*JAMIA Open*] online.

### 5.2 Baseline models

We compared our model against ARIMA,^18^ Random Forest Regression (RFR),^19^ LSTM,^20^ and GConvLSTM;^21^ details are listed in Appendix B, available as supplementary data^22^ at [*JAMIA Open*] online.

## 6 Experiment

To evaluate the effectiveness of the proposed method, we investigate the following research questions through a series of experiments. Implementation details are provided in Appendix C, available as supplementary data at [*JAMIA Open*] online.

**Q1:** Does the theoretical analysis data assumption hold?

**Q2:** How does population size affect model performance?

**Q3:** How effective are the proposed AAE-STGNN in predicting OD death?

### 6.1 Q1: does the theoretical analysis data assumption hold?

**Figure 4.**
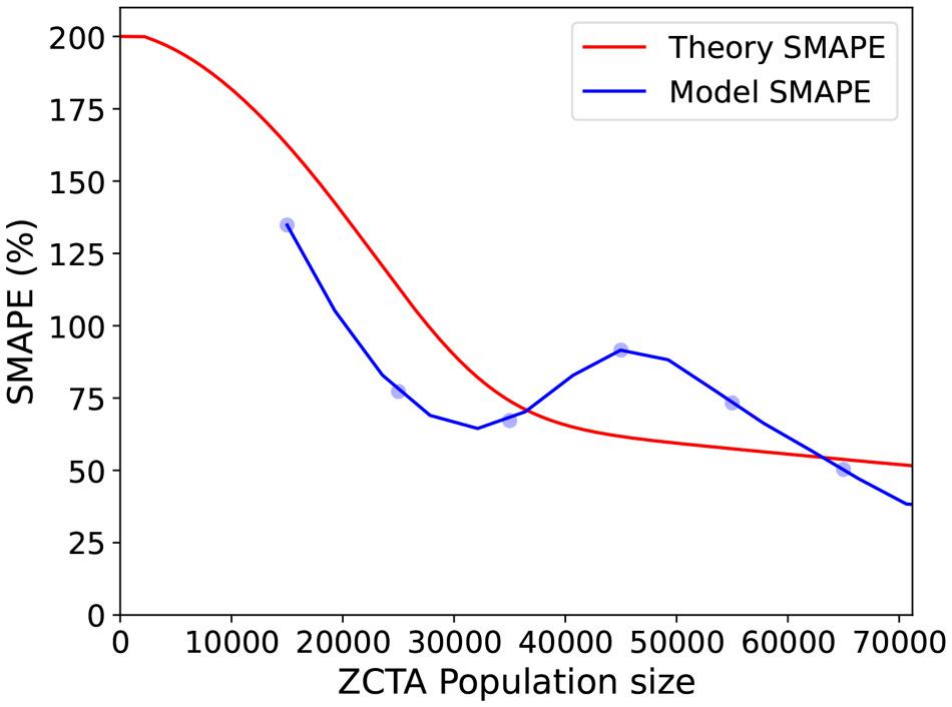
Theoretical SMAPE vs the SMAPE of model predictions.

As shown in Figure 4, the SMAPE of the model predictions closely aligns with the theoretical SMAPE. This indicates that our assumptions are reasonable: for large counties, the OD counts approximately follow a Normal(*np*, *np*) distribution, while for small counties, the Bernoulli(*np*) approximation is appropriate. By validating these data assumptions, our analysis increases confidence in the reliability of model outputs, which is essential for supporting informed decision-making.

### 6.2 Q2: how does population size affect model performance?

To further investigate the impact of population size on predictive accuracy, we evaluated the performance of AAE-STGNN across counties grouped by population size intervals. Specifically, counties were stratified into four population bins: 10k–20k, 20k–30k, 30k–50k, and above 50k. As shown in Table 1, prediction error decreases consistently as population size increases. Counties in the smallest population bin (10k–20k) exhibit substantially higher SMAPE, reflecting higher relative noise and instability in sparse populations. As population size increases, SMAPE decreases monotonically, indicating improved robustness and signal reliability. A similar trend is observed for MAE and RMSE, with larger counties achieving more accurate absolute predictions.

**Table 1.** Performance comparison of AAE-STGNN across county population size groups.

County	SMAPE	MAE	RMSE	Mean	GT	Prediction
Size	(%)			Population		
10k–20k	141.32	0.30	0.44	15,090	0.26	0.25
20k–30k	96.49	0.42	0.55	26,736	0.57	0.48
30k–50k	80.29	0.45	0.55	39,390	0.62	0.69
50k+	57.89	2.55	5.85	214,554	5.82	4.64

For all metrics, lower values indicate better performance (↓).

*Note.* GT denotes the average ground-truth overdose death counts, and Prediction denotes the average model-estimated counts within each population size group. Mean Population refers to the average county population in each bin.

These results empirically support our earlier signal-to-noise analysis: population size plays a critical role in stabilizing model performance. In particular, performance gains become less pronounced beyond a population size of approximately 30,000, suggesting that prediction error has largely stabilized at this scale. Overall, these findings indicate that aggregating data at appropriately sized county-level units effectively balances noise reduction and local interpretability, providing a principled foundation for reliable overdose death prediction and downstream public health decision support.

### 6.3 Q3: how effective are the proposed AAE-STGNN in predicting OD death?

#### 6.3.1 Overall performance comparison

The primary goal of this study is to accurately predict OD death counts. To evaluate the effectiveness of the proposed framework, we conduct comparative experiments against several baseline models described in subsection 5.2.

Table 2 summarizes the predictive performance of our proposed framework (AAE-STGNN) and several baselines across multiple evaluation metrics. Our approach consistently achieves the best performance, substantially outperforming all baselines. Specifically, compared with the strongest baseline, GConvLSTM, our overall model which includes all counties achieves a 15.70% relative reduction in SMAPE (from 78.00% to 62.30%), indicating more accurate trend prediction across counties. In terms of MAE, AAE-STGNN reduces the average absolute prediction error from 1.97 to 0.86, which corresponds to approximately 1.11 fewer deaths per county per quarter. Furthermore, RMSE decreases from 5.49 to 1.59, demonstrating that the fluctuation of prediction errors is reduced by about 3–4 deaths, meaning that extreme deviations in predicted death counts are much less pronounced. While GConvLSTM produces competitive results, it remains less effective in capturing complex spatiotemporal dependencies. Overall, these results confirm that the proposed spatiotemporal modeling framework provides more accurate and robust county-level OD death predictions, with strong potential to support decision-making via a public health dashboard for the Ohio Department of Health, enabling timely situational awareness and proactive planning.

**Table 2.** Main result of different county-level OD quarter prediction.

Method	SMAPE(%)	MAE	RMSE
ARIMA	93.02	1.36	3.41
RFR	97.02	2.32	5.45
LSTM	85.99	2.13	5.69
GConvLSTM	78.00	1.97	5.49
AAE-STGNN	**62.30**	**0.86**	**1.59**

For all metrics, lower values indicate better performance (↓). The best result is marked in bold.

Additionally, to provide public health decision-makers with a more diverse set of tools, our framework not only predicts the absolute number of OD deaths, but also forecasts the quarterly trend. Identifying whether deaths are expected to decrease, remain stable, or increase provides actionable information for early intervention and resource allocation, complementing raw count forecasts by highlighting potential emerging hotspots. The dataset exhibits class imbalance: increasing, stable, decreasing trends account for approximately 31.87%, 31.55%, and 36.59% respectively. In this multi-class setting, we report accuracy, precision, recall, and F1.

As shown in Table 3, our proposed method, AAE-STGNN, consistently outperforms baseline approaches across all evaluation metrics. Beyond aggregate performance, AAE-STGNN demonstrates clear advantages in class-wise trend prediction, particularly for the stable and decreasing trend classes, where it yields higher accuracy than all baseline methods. Although the accuracy of the stable class remains low across all methods, this is expected given the strict definition of stability in our setting: only counties with completely unchanged outcomes are labeled as stable, while many predictions correspond to counties exhibiting minor fluctuations. This challenge is further illustrated by the LSTM baseline, which attains zero accuracy for the stable class, leading to particularly low precision. Overall, these results highlight the superior reliability of AAE-STGNN in capturing meaningful OD trends under realistic and imbalanced conditions. Accurate identification of all three classes is critical for decision-making, as it enables early prioritization of public health resources toward counties with worsening trends.

**Table 3.** Performance comparison of different methods for county-level OD trend prediction.

Method	Acc	Prec	Rec	F1	I-Acc	S-Acc	D-Acc
ARIMA	0.3667	0.3985	0.3566	0.2983	0.5037	0.0176	0.5483
RFR	0.3540	0.3682	0.3432	0.2816	0.4751	0.0050	0.5494
LSTM	0.3723	0.2484	0.3625	0.2944	**0.5076**	0.0000	0.5440
GConvLSTM	0.3730	0.3484	0.3656	0.3005	0.4877	0.0100	0.5640
AAE-STGNN	**0.3826**	**0.4392**	**0.3720**	**0.3178**	**0.5076**	**0.0340**	**0.5744**

The three trend classes are decreasing, stable, and increasing. The best result for each metric is marked in bold.

*Note.* Acc, Prec, and Rec denote accuracy, precision, and recall, respectively. I-Acc, S-Acc, and D-Acc denote class-wise accuracy for the increasing, stable, and decreasing trend classes.

**Figure 5.**
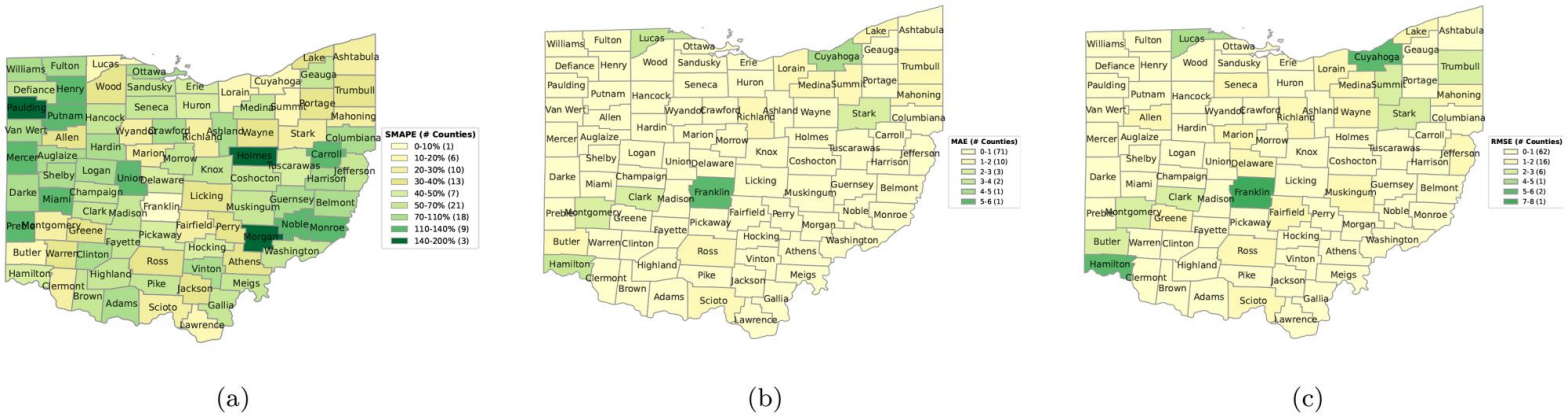
Visualization of county-level performance metrics in Ohio: (a) SMAPE, (b) MAE, and (c) RMSE.

As shown in Figure 5, we present the SMAPE, MAE, and RMSE of each Ohio county over the test period. Lower values indicate better predictive performance, with lighter colors representing lower errors on the maps. For SMAPE, large counties such as Franklin County (population 1,290,360) and Summit County (population 541,334) exhibit relatively low errors, whereas smaller counties, such as Paulding County (population 18,809), show higher SMAPE values due to their smaller populations, reflected by darker colors. For MAE and RMSE, a similar pattern emerges: smaller counties tend to have lower absolute errors because the baseline counts are small, making deviations numerically limited, while larger counties show relatively higher MAE and RMSE despite good relative performance. This spatially explicit error characterization enhances interpretability and provides practical guidance for decision makers, supporting the use of a county-level public health dashboard for the Ohio Department of Health to assess prediction reliability and prioritize attention across regions.

#### 6.3.2 Ablation study

To evaluate the contribution of the data augmentation stage in the proposed method, we conduct an ablation study as shown in Table 4. Without the data augmentation component, the model (STGNN) performs worse than the full version (AAE-STGNN), highlighting the importance of the area-specific autoencoder in enhancing feature representation learning.

**Table 4.** Performance comparison between with and without data augmentation.

Method	SMAPE(%)	MAE	RMSE
STGNN	76.53	1.00	2.69
AAE-STGNN	62.30	0.86	1.59

## 7 Conclusion

In this work, our aims were to identify the optimal spatial level for OD prediction and to develop an effective model that integrates UDT and SDoH data. Through an SNR analysis, we found that regions with populations smaller than 30,000 exhibit excessive variability in death counts, leading to high noise and unstable predictions. Based on this analysis, we proposed a new framework, AAE-STGNN, which first applies a population threshold of 30,000 to aggregate neighboring areas, allowing the model to learn meaningful spatiotemporal representations. Then, a STGNN was employed to incorporate static SDoH features for county-level OD predictions. Experiments on real-world Ohio data demonstrated that AAE-STGNN achieves accurate and reliable OD prediction.

As a next step, this algorithm is planned to be integrated into a public health dashboard on the Ohio Department of Health website, providing decision-makers with actionable insights for proactive planning and resource allocation.

## Supplementary Material

ooag063_Supplementary_Data

## Data Availability

The source code for the proposed framework is publicly available on GitHub (https://github.com/XianhuiChen/AAE_STGNN). SDoH data are publicly accessible from the U.S. Census Bureau’s American Community Survey (ACS). Access to individual-level UDT data requires appropriate data use agreements and can be requested from the corresponding author in collaboration with Millennium Health staff.
