# Supplementary material for: Area-specific autoencoder spatiotemporal graph neural networks for opioid overdose death prediction
Source: JAMIA Open. 2026 Apr 29;9(2):ooag063. doi: 10.1093/jamiaopen/ooag063 (PMC13127421; doi:10.1093/jamiaopen/ooag063)
Supplement: ooag063_Supplementary_Data [file ooag063_supplementary_data.zip › Manuscript_clean_Part2.pdf]

A METRIC

SMAPE: Captures relative error and is scale-invariant, making it suitable for comparing prediction accuracy across regions with different population sizes. SMAPE reflects the relative discrepancy between predicted and observed deaths. It is more informative for larger counties where relative error matters, but can be unstable for areas with very small counts, potentially exceeding 200% for tiny populations—hence care must be taken when interpreting SMAPE in low-count regions. The equation of SMAPE for a single area i is shown in Equation 5. The overall SMAPE across all N areas is then defined as:

$$\text{SMAPE} = \frac{1}{N}\sum_{i=1}^{N}\text{SMAPE}_i = \frac{1}{N}\sum_{i=1}^{N}\frac{2|y_i - \hat{y}_i|}{|y_i| + |\hat{y}_i|}, \quad (26)$$

where y_i is the observed count and $\hat{y}_i$ is the predicted count for area i.

Mean Absolute Error (MAE): Measures the average magnitude of prediction errors and reflects the absolute deviation between predicted and observed OD death counts. MAE is straightforward to interpret and is particularly informative for areas with small populations, where absolute differences directly indicate under- or overestimation of deaths. Formally, for N areas:

$$\text{MAE} = \frac{1}{N}\sum_{i=1}^{N}|y_i - \hat{y}_i|, \quad (27)$$

where y_i is the observed count and $\hat{y}_i$ is the predicted value for area i.

Root Mean Squared Error (RMSE): Measures the square root of the average squared prediction errors, giving greater weight to larger errors. RMSE is especially useful for smaller regions where large deviations are more consequential, as it penalizes outlier predictions more heavily than MAE. Formally, for N areas:

$$\text{RMSE} = \sqrt{\frac{1}{N}\sum_{i=1}^{N}(y_i - \hat{y}_i)^2}, \quad (28)$$

where y_i is the observed count and $\hat{y}_i$ is the predicted value for area i.

B BASELINE MODELS

- **ARIMA (Autoregressive Integrated Moving Average):**[18] A classical statistical model for time-series forecasting that captures temporal autocorrelations through autoregressive and moving average components. ARIMA is particularly suitable for OD death counts prediction over time, providing a strong baseline for comparison with machine learning models on temporal dynamics.
- **Random Forest Regression (RFR):**[19] An ensemble learning method using multiple decision trees for regression, averaging their predictions to improve accuracy and reduce overfitting.
- **LSTM:**[20] Standard sequence model for temporal dynamics.
- **GConvLSTM (Graph Convolutional LSTM):**[21] Extends LSTM by incorporating graph convolutions for spatial context.

C IMPLEMENTATION DETAILS

The data are split chronologically by quarter into training, validation, and testing sets, corresponding to approximately 70%, 10%, and 20% of the data, respectively. The maximum number of training epochs for all networks is set to 300. The initial learning rate η is empirically chosen as 0.003, and optimization is performed using the Adam optimizer[22]. The observation window is set to three quarters. All hyperparameters are selected via grid search.

Regions with very few individuals can lead to noisy labels. Therefore, we reduce noise by randomly combining multiple ZCTAs in the training set to ensure that the population exceeds a certain threshold. All corresponding data—including SDoH scores, UDT —are merged accordingly (count-type data are summed, percentage-type data are averaged). This process effectively constructs a new "virtual" sample for training. For the validation and test sets, we use the original data.

D DATA

D.0.1 Outcome Data

Quarterly counts of OD death counts were obtained from the National Vital Statistics System (NVSS) multiple cause-of-death files. Following standard surveillance definitions, opioid-involved overdose deaths were identified using ICD-10 underlying cause-of-death codes X40–X44 (unintentional), X60–X64 (suicide), X85 (homicide), Y10–Y14 (undetermined intent), and T40.0–T40.4, T40.6 (opioid poisoning). These OD death counts were extracted for each quarter from Q1 2013 through Q4 2023.

D.0.2 Exposure Data

Our exposure features draw from two major data domains: UDT and SDoH.

Table 5. Overview of features used for OD death prediction, derived from UDT data collected between Q1 2013 and Q4 2023.

Data	Feature
ODH Vitals	Drug OD deaths
UDT	Cocaine: test count, positive count, and positive rate
UDT	Fentanyl: test count, positive count, and positive rate
UDT	Heroin: test count, positive count, and positive rate
UDT	Heroin and morphine: test count, positive count, and positive rate
UDT	Methamphetamine: test count, positive count, and positive rate
UDT	Stimulant (methamphetamine and/or cocaine): test count, positive count, and positive rate
UDT	F_M: test count, positive count, and positive rate
UDT	F_C: test count, positive count, and positive rate
UDT	F_S: test count, positive count, and positive rate
UDT	F_H: test count, positive count, and positive rate
UDT	Benzodiazepines: test count, positive count, and positive rate
UDT	Prescription opioids (hydrocodone, hydromorphone, oxycodone, oxymorphone, codeine): test count, positive count, and positive rate
UDT	Polysubstance use: Positive when ≥ 2 different substances are detected in the same UDT sample (any combination from the full drug panel). Negative if all tests are negative or only one substance is positive.
UDT	Alcohol: test count, positive count, and positive rate
ACS	SDI score
ACS	Percentage of poverty below 100% Federal Poverty Level
ACS	Percentage of single-parent families
ACS	Percentage of low education attainment (adults with less than 12 years of education)
ACS	Percentage of households without a vehicle
ACS	Percentage of renter-occupied households (rather than owner-occupied)
ACS	Percentage of household crowding (more than one person per room)
ACS	Percentage of non-employed individuals

Note: OD = opioid overdose; UDT = urine drug test; ACS = American Community Survey; SDI = Social Deprivation Index. For UDT-derived features, "test count" = number of tests conducted; "positive count" = number of positive tests; "positive rate" = proportion of positive tests. F_M = Methamphetamine among fentanyl-positive samples, F_C, F_S, F_H = Cocaine, Stimulants, and Heroin among fentanyl-positive samples, respectively.

1. **UDT:** We used aggregated quarterly UDT data from Millennium Health's national testing database, which includes specimens collected from adults (≥18 years) across all 88 counties in Ohio. Tests were collected between Q1 2013 and Q4 2023 and analyzed using validated LC-MS/MS methods under Clinical Laboratory Improvement Amendments (CLIA) and College of American Pathologists (CAP) accreditation. For each quarter, we computed drug-specific positivity rates based on the number of positive tests and total tests, capturing the prevalence of substance use and co-use. Quarters with no received tests were treated as missing and filled with zeros, reflecting the absence of observed tests rather than absence of substance use. These aggregated UDT measures were used as a proxy for substance use patterns, enabling estimation of temporal and geographic trends in opioid exposure.

   UDT data are not obtained through random population sampling but arise from clinical testing of individuals based on medical necessity, including to meet requirements of substance use treatment programs and when a medical provider suspects substance use. In our current study, all UDT specimens were sampled from substance use treatment clinics. As a result, UDT signals in our sample reflect a risk-enriched subpopulation, shaped by people with both confirmed and suspected history of substance use, and, thus, is likely to confer greater information about the population at-risk of overdose. While such a selection bias is a limitation when conducting inferential research, a data source which over-represents the population of people who use drugs should theoretically have greater overdose prediction utility than a data source which generalizes to the full population of Ohio. This is supported by prior research which has evaluated the utility of UDT for overdose prediction.

2. **SDoH:** To capture contextual community risk factors, we included county-level Social Deprivation Index (SDI), socioeconomic variables to measure: poverty, unemployment rates, educational attainment, and health-care access. These measures represent structural determinants associated with spatial disparities in overdose risk.

The details of the features are listed in Table 5.

E MODEL CONFIGURATION

A detailed description of the model configuration is presented in Table 6.

Table 6. Detailed configuration of AAE-STGNN.

Block	Layer Configuration
**Temporal Encoder**	LSTM (hidden dim = 64, layers = 1)
**Static Fusion**	Concatenate h_T with static area-level features<br>Fully Connected Layer (64), ReLU
**Graph Encoder**	GCNConv (64), ReLU<br>GCNConv (1)
**Pooling**	Global Mean Pooling over nodes within each area<br>Output reshaped to area-level scalar representation

F TEMPORAL ROBUSTNESS ANALYSIS

As shown in Table 7, the predictive performance of AAE-STGNN is highly consistent across years. SMAPE varies within a narrow range (62.02–62.69%), and both MAE and RMSE increase gradually but remain comparable across 2021–2023. This temporal stability suggests that the model effectively captures persistent spatiotemporal patterns rather than overfitting to year-specific signals, and that its performance is minimally affected by temporal non-stationarity.

Table 7. Year-wise prediction performance of AAE-STGNN. Lower values indicate better performance (↓).

Year	SMAPE (%)	MAE	RMSE
2021	62.29	0.77	1.15
2022	62.02	0.86	1.51
2023	62.69	0.98	2.03
